# Epizootic Hemorrhagic Disease in Cattle, Western Turkey

**DOI:** 10.3201/eid1502.080572

**Published:** 2009-02

**Authors:** Ethem Mutlu Temizel, Kadir Yesilbag, Carrie Batten, Sezgin Senturk, Narender S. Maan, Peter Paul Clement Mertens, Hasan Batmaz

**Affiliations:** Uludag University Faculty of Veterinary Medicine, Gorukle, Turkey (E.M. Temizel, K. Yesilbag, S. Senturk, H. Batmaz); Institute for Animal Health, Woking, UK (C. Batten, N.S. Maan, P.P.C. Mertens)

**Keywords:** epizootic, clinical signs, outbreak, cattle, PCR, ELISA, Turkey, dispatch

## Abstract

In 2007, an outbreak of epizootic hemorrhagic disease (EHD) occurred in Turkey. On the basis of clinical investigation, 41 cattle were suspected to have EHD. Reverse transcription–PCR and sequence analyses indicated that the virus belonged to EHD virus serotype 6, thus confirming EHD virus infection of cattle in Turkey.

Epizootic hemorrhagic disease virus (EHDV) is a member of the genus *Orbivirus*, family *Reoviridae,* and is closely related to bluetongue viru*s* (BTV). EHD often causes death in white-tailed deer and, less frequently, a bluetongue-like illness in cattle (*1*–*3*).

*Culicoides* spp. act as vectors, transmitting EHDV between susceptible ruminant hosts (*2*). The clinical signs of EHD in cattle are fever, anorexia, dysphagia, ulcerative and necrotic lesions of the oral mucosa, hyperemia and edema of the conjunctival mucosae, sore muzzle, hyperemia of the teats and udder, hemorrhage, dehydration, and lameness (*3*). EHDV has been isolated from cattle throughout the world, including Africa, North America, Australia, Japan, and recently Israel (*4*–*10*). Recent outbreaks of EHDV in Israel during 2006 were attributed to EHDV-7 (*6*); outbreaks in Morocco and Algeria were similar to EHDV-6/EHDV-318. An initial suspicion of EHD, based on observation of clinical signs, can be confirmed by virus isolation and characterization, nucleic acid identification, or serologic testing. ELISA is a specific and sensitive method for detecting EHDV-specific antigens or antibodies and confirming the disease (*2*–*5*,*8*,*11*,*12*).

## The Study

In July 2007, a 7-week outbreak of disease in cattle began in Mugla, Turkey. The disease was regarded as unusual or atypical for the region, and cases were reported to the Uludag University Faculty of Veterinary Medicine. Similar reports were also received from Izmir, Canakkale, and Istanbul through the end of August 2007. The cattle had stomatitis, swelling of eyelids, respiratory distress, nasal and ocular discharge, redness and scaling of muzzle and lips, lameness, and udder erythema, and some were recumbent (Table 1). Body temperatures were elevated (39.7°C–41.1°C ), except for 1 animal, whose temperature was 37.5°C, below the reference range for cattle (37.8°C–39.2°C). However, heart rates (mean 72 ± 3 beats/min) and respiratory rates (mean 24 ± 4 breaths/min) were within reference ranges of 60–80 beats/min and 10–30 breaths/min, respectively, for cattle with suspected disease. Cattle with EHD had tachycardia and tachypnea (Table 2). Causes of mucosal disease, stomatitis, and fever, including bovine viral diarrhea, foot and mouth disease, and infectious bovine rhinotracheitis, were considered, but the rate of spread and some of the clinical signs ruled out these diseases. However, the clinical signs of the disease were consistent with either EHD or BTV infection (*6*,*8*–*10*). These diseases were therefore considered as requiring further laboratory-based diagnostic assays.

**Table 1:** Clinical signs in cattle tested for EHD, Turkey, 2007*

EHD status†	No. cattle with clinical sign									
	Discharge‡	Redness§	Recumbency	CE	Anorexia	RM	UE	Stomatitis	RD	Lame
Suspected (n = 41)	13	12	2	15	16	20	9	12	5	6
PCR+ (n = 1)	1	0	1	1	1	1	1	1	1	1
Seropositive (n = 1)	1	1	0	1	1	1	0	1	0	1
Virus isolated (n = 6)	5	6	0	4	6	6	0	4	3	3

*EHD, epizootic hemorrhagic disease; CE, conjunctival edema; RM, reduced milk; UE, udder edema; RD, respiratory distress. †PCR, ELISA, and virus isolation were performed on selected samples from the 41 samples (11 whole blood samples, 4 serum samples, and 15 supernatant samples from the baby hamster kidney cells). The virus-positive animals were PCR negative. ‡Nasal and ocular discharge §Redness and scaling of nose and lips.

**Table 2:** Vital signs of cattle tested for EHD, Turkey, 2007*

EHD status†	Temperature, °C	Heart rate, beats/min	Respiratory rate, breaths/min	Mucous membranes	Enlarged lymph nodes	Rumen motility, contractions/5 min
Suspected (n = 41)	37.5–39.2	72 ± 3‡	24 ± 4‡	Cyanotic (n = 2)	ND	0–12
PCR+ (n = 1)	40.5	110	52	Cyanotic	Prescapular, submandibular	0
Seropositive (n = 1)	41.1	104	48	Hyperemic	None	1
Virus isolated (n = 6)	39.7–40.6	68–86	32–56	Normal color	Submandibular (n = 1)	0–4

*EHD, epizootic hemorrhagic disease; ND, not detected. †PCR, ELISA, and virus isolation were performed on selected samples from the 41 samples (11 whole blood samples, 4 serum samples, and 15 supernatant samples from the baby hamster kidney cells). The virus-positive animals were PCR negative. ‡Mean, ± SEM.

A total of 41 blood samples were obtained from the affected cattle (35 Holsteins and 6 Brown Swiss, 2–5 years of age). Samples were obtained in tubes with and without EDTA. Complete blood analysis showed that 5 of the cattle with EHD had low leukocyte counts (Appendix Table). After use for hematologic analysis, samples were stored at –30°C until virologic and serologic tests could be performed. Samples from the 41 animals were tested by ELISA for bovine viral diarrhea virus antigens; results were negative. To isolate virus, we spread unclotted blood samples onto baby hamster kidney–21 (BHK) cells.

Because EHDV had never been observed in Turkey, no diagnostic procedures were available. We therefore submitted selected samples (11 whole blood samples, 4 serum samples, and 15 supernatant samples from the BHK cells) to the World Organisation for Animal Health reference laboratory for BTV (Institute for Animal Health, Pirbright, UK) for virologic and serologic analysis. All samples were tested for BTV by real-time RT-PCR and for EHDV by conventional RT-PCR (*13*–*15*). All results were negative for BTV. However, a conventional RT-PCR assay targeting genome segment 7 of EHDV (*15*) indicated that one of the cell culture supernatants, from an early case from Mugla, was positive for EHDV; this cow died 3 hours after clinical examination and sample collection. The remaining cell culture supernatants were negative for EHDV. It is unusual to isolate EHDV by direct inoculation of BHK cells; initial passage through eggs or the *Culicoides variipennis* larvae cell line (KC cells) is usually required (*15*). The 4 serum samples were also tested for EHDV-specific antibodies by ELISA (*12*); only 1 sample was found to contain antibodies to EHDV.

Conventional RT-PCR of RNA extracted from the 11 original blood samples gave inconclusive results. Agarose gel electrophoresis indicated no product of the expected size. However, virus was isolated from 6 of the blood samples by using KC cells (dsRNA virus reference collection at the Institute for Animal Health, reference collection nos. TUR2007/01-06). These 6 samples and the 1 original positive cell culture were further tested by serotype-specific RT-PCRs that targeted segment 2 for identification of EHDV serotype. This analysis identified all viruses as EHDV-6, sharing 95.7% nucleotide sequence identity (segment 2, 110–670 bp) with the EHDV reference strain 318.

## Conclusions

Of the selected samples submitted for BTV and EHDV testing, the positive identification of EHDV RNA supports initial clinical identification of an EHD outbreak in Turkey. The negative results from the blood samples may have resulted from degradation of viral RNA during transfer to the laboratory or insufficient sensitivity in the conventional RT-PCR. The propagation of another 6 virus isolates (TUR2007/01–06) by passage through KC cells indicates that virus was indeed present in the original blood samples, although not detected by conventional RT-PCR.

That only 1 of the 4 original serum samples was positive for EHDV antibodies by ELISA can be explained by time of sample collection. Antibodies to BTV can be detected from 8 days after infection (*11*); these samples may have been collected during the early stages of infection, before development of the immune response.

This study confirms EHDV infection of cattle in Turkey. EHD needs to be considered in the differential diagnosis of cattle with clinical signs that include fever; stomatitis; lameness; salivation; redness and scaling of the nose and lips; swelling of the tongue; and erosions of the pulvinus dentalis, palatinum, and nose. More detailed studies of EHDV infection in cattle are needed.

**Figure 1:**
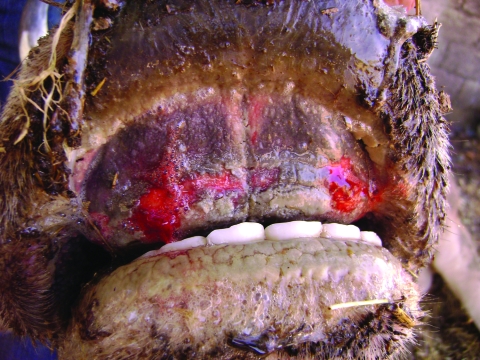
Erosive lesion on pulvinus dentalis of cow seropositive for epizootic hemorrhagic disease virus, Turkey, 2007**.**

**Figure 2:**
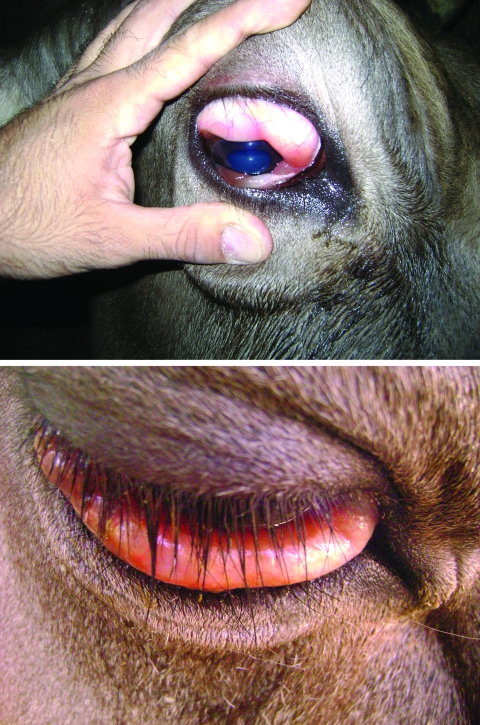
Swollen conjuctiva of cow seropositive for epizootic hemorrhagic disease virus, Turkey, 2007**.**

## Supplementary Material

Appendix Table Hematologic findings in cattle tested for epizootic hemorrhagic disease, Turkey, 2007*
