# Supplementary material for: Epizootic Hemorrhagic Disease in Cattle, Western Turkey
Source: Emerg Infect Dis. 2009 Feb;15(2):317–9. doi: 10.3201/eid1502.080572 (PMC2662652; doi:10.3201/eid1502.080572)
Supplement: Appendix Table — Hematologic findings in cattle tested for epizootic hemorrhagic disease, Turkey, 2007* [file 08-0572_appT-s1.pdf]

Publisher: CDC; Journal: Emerging Infectious Diseases
Article Type: Dispatch; Volume: 15; Issue: 3; Year: 2009; Article ID: 08-0572
DOI: 10.3201/eid1502.080572; TOC Head: Dispatch

Appendix Table. Hematologic findings in cattle tested for epizootic hemorrhagic disease, Turkey, 2007*

EPH status†	WBCs, ×10^3/µL	Neutrophils, %	Lymphocytes, %	Monocytes, %	Eosinophils, %	Basophils, %	RBCs, ×10^6/µL	HGB, g/dL	HCT, %	MCV, µm^3	MCH, pg/cell	MCHC, g/dL	RDW, %	PLT, ×10^3/µL
Suspected (n = 41) ‡	4.3 ± 0.3	55.1 ± 2.7	33.2 ± 2.8	9.1 ± 0.8	0.3 ± 0.1	1.6 ± 0.3	6.4 ± 0.2	9.2 ± 0.2	25.7 ± 0.7	40.8 ± 0.9	15 ± 0.5	36.7 ± 0.8	22.9 ± 0.4	359.4 ± 32
PCR+ (n = 1)	1.99	66.5	22.8	8.59	0.224	1.82	5.42	9.27	24.8	45.8	17.1	37.3	20	168
Seropositive (n = 1)	5	30	56	6	8	0.8	6.43	8.5	24	41	13.4	35.2	19.8	150
Virus isolated (n = 6)	1.6–5.13	36.2–70.3	14.6–59.2	3.03–23.5	0–2.5	0.48–4.2	3.9–6.7	7.0–11.8	17.8–29.9	45.1–48.8	17.2–19.9	37.5–40.7	18.9–20.9	152–603

*EPH, epizootic hemorrhagic disease; WBCs, white blood cells; RBCs, red blood cells; HGB, hemoglobin; HCT, hematocrit; MCV, mean corpuscular volume; MCH, mean corpuscular hemoglobin; MCHC, mean corpuscular hemoglobin concentration; RDW, red blood cell distribution width; PLT, platelets.
†PCR, ELISA, and virus isolation were performed on selected samples from the 41 samples (11 whole blood samples, 4 serum samples, and 15 supernatant samples from the baby hamster kidney cells). The virus-positive animals were PCR negative.
‡Mean ± standard error of the mean.
